# Freeze–Thaw Resistance and Air-Void Analysis of Concrete with Recycled Glass–Pozzolan Using X-ray Micro-Tomography

**DOI:** 10.3390/ma14010154

**Published:** 2020-12-31

**Authors:** Marija Krstic, Julio F. Davalos, Emanuele Rossi, Stefan C. Figueiredo, Oguzhan Copuroglu

**Affiliations:** 1 Department of Civil Engineering, City University of New York (City College), 160 Convent Avenue, New York, NY 10031, USA; jdavalos@ccny.cuny.edu; 2 Microlab, Department of 3MD, Faculty of Civil Engineering and Geosciences, Delft University of Technology, Stevinweg 1, 2628 CN Delft, The Netherlands; e.rossi@tudelft.nl (E.R.); S.ChavesFigueiredo@tudelft.nl (S.C.F.); o.copuroglu@tudelft.nl (O.C.)

**Keywords:** cementitious materials, glass pozzolan GP, freeze–thaw resistance, dynamic modulus of elasticity, MIP, X-ray micro-tomography, air void analysis

## Abstract

Recent studies have shown promising potential for using Glass Pozzolan (GP) as an alternative supplementary cementitious material (SCM) due to the scarcity of fly ash and slag in the United States. However, comprehensive studies on the freeze–thaw (FT) resistance and air void system of mixtures containing GP are lacking. Therefore, this study aimed to evaluate GP’s effect on FT resistance and characterize mixtures with different GP contents, both macro- and microscopically. In this study, six concrete mixes were considered: Three mixes with 20%, 30% and 40% GP as cement replacements and two other comparable mixes with 30% fly ash and 40% slag, as well as a mix with 100% Ordinary Portland cement (OPC) as a reference. Concrete samples were prepared, cured and tested according to the ASTM standards for accelerated FT resistance for 1000 cycles and corresponding dynamic modulus of elasticity (Ed). All the samples showed minimal deterioration and scaling and high F/T resistance with a durability factor of over 90%. The relationships among FT resistance parameters, air-pressured method measurements of fresh concretes and air void analysis parameters of hardened concretes were examined in this study. X-ray micro-tomography (micro-CT scan) was used to evaluate micro-cracks development after 1000 freeze–thaw cycles and to determine spatial parameters of air voids in the concretes. Pore structure properties obtained from mercury intrusion porosimetry (MIP) and N_2_ adsorption method showed refined pore structure for higher cement replacement with GP, indicating more gel formation (C-S-H) which was verified by thermogravimetric analysis (TGA).

## 1. Introduction

The improvement of durability properties of concrete materials deserves special attention in order to extend the service life of structures. Besides mechanical loads, concrete structures are exposed to environmental effects as well (e.g., low-temperature weather conditions) and they can be harmful for porous brittle materials such as concrete. When concrete is subjected to repetitive freezing and thawing (FT) cycles, its durability is affected, leading to accelerated deterioration and loss of stiffness and strength. Freezing and thawing resistance is an essential durability property of concrete in cold weather environments. The water starts to freeze in capillary pores at temperatures well below the freezing point. When water turns into a solid state, the volume of ice is greater than the volume of the pore water and induces an expansion of about 9% [1,2,3]. Considering the freeze–thaw phenomenon, the most important factor in air void properties is pore interconnectivity. In normal concrete the capillary pores (usually between 5 nm and 1 mm) are responsible for creating a network of voids. Capillary forces in such small volumes are very important in order to allow the water transport inside the paste matrix [1]. One of the severe types of deterioration in concrete structures is associated with the volume expansion of water caused by freezing and thawing [2]. This volume expansion results in pressure build-up inside the pores if not accommodated with sufficient pore space and inter-connectivity in the matrix [3]. If the pressure exceeds the tensile strength of the cement paste at any point it will lead to local cracking, hence the strength of concrete will decrease after a number of FT cycles [4]. The magnitude of this induced hydraulic pressure depends on the permeability of the cement paste, the degree of saturation, the distance of the nearest unfilled void and the rate of freezing [5].

One of proven methods to improve the FT resistance is to add air-entraining agents. Regular concrete contains about 2% to 3% of entrapped air (air bubbles are accidental due to mixing and they could range between 0.3 and 5 mm). In contrast, entrained air bubbles are intentional and they range from 1 to 100 µm [6]. Besides increased air void content, it is believed that for freeze–thaw resistant concrete it is necessary to have a spacing factor smaller than 0.2 mm (200 µm) and specific surface of air void system greater than 24 mm^2^/mm^3^ [7,8]. Air void analysis is usually performed as conventional method ASTM C457. The method requires a tedious preparation of large samples dependent on aggregate size. Samples are viewed in 2D through optical microscope or flatbed scanner [9,10]. Then standardized stereological method is applied (linear traverse method or point counting). Besides ASTM C457, X-ray computed tomography (CT) was also applied to characterize the air void system of cementitious materials [11]. X-ray computed tomography is found to be a nondestructive method that uses high resolution for characterization of materials in 3D. It does not require a long sample preparation. The limitation of this method is the size of samples that can fit into the machine and still accomplish an appropriate resolution in order to see the air voids [12,13].

Cement based concrete is the most used composite material in the world for building structures, due to its versatility, durability and favorable cost [14]. Nowadays, however, the concrete construction industry faces a big challenge in regard to sustainability criteria. The production of cement is an energy intensive process, where about half of the CO_2_ emissions originate from the CaCO_3_ calcination, while the remaining carbon is from the energy used during this process [15,16]. The cement manufacturing has raised environmental concerns since one ton of cement produces nearly one ton of CO_2_ (5–8% contribution to total global CO_2_ emissions) [17,18,19]. In the USA about 90 million tons of cement are used annually (CO_2_ emissions equivalent to 300 million cars) [18]. To overcome environmental impacts and produce high-performance concretes (HPC), there is a significant interest in using recycled materials. Commonly, supplementary cementitious materials (SCMs) are byproducts, such as fly ash (from burning pulverized coal in electric generating plants) and slag (from steel production), used today to partially replace cement content. However, due to recent environmental protection regulations, the availability of fly ash in the USA has decreased significantly [20,21,22]. The granulated blast-furnace slag is relatively expensive and generally produced outside the USA (Japan 33%, Canada 31%, Spain 16%, Germany 5% and other 15%) [23]. The scarcity and cost of SCMs in the USA is of concern to the concrete industry. Thus, there is a need for an alternate SCM to overcome the dwindling supply of fly ash, particularly in the Northeastern region of the USA, and recycled post-consumer soda-lime glass has received increased attention in the concrete industry in recent years, since it can be effectively and economically transformed into value-added pozzolanic material for concrete [24]. In New York state, 3 million tons of cement are used annually for concrete [18], and assuming an overall 30% SCM cement replacement, there is a potential market for one million tons per year of glass pozzolan (GP) (resulting in nearly one million tons of CO_2_ reduction) [25]. Glass Pozzolan is an inert material that when milled to micro-level particles does not change its chemical composition and provides favorable pozzolanic reactivity [17,26].

Since the application of GP has high potential to be largely used as SCM, it is very important to evaluate its functional and durability-related properties. Among others, only limited studies [27] have evaluated the freeze–thaw resistance of concrete with GP replacements. Therefore, the goal of this study is to establish correlations among freeze–thaw resistance, pore structure and air void parameters for concretes with different GP cement replacements. After the macroscopic characterizations were completed, internal micro-crack studies and air void analyses were conducted using X-ray micro-tomography (CT-scan), in order to correlate macro and micro-evaluations. The findings in this study provide a multiscale understanding of durability properties of concrete with GP as SCM, and the results contribute to practical implementations in cold environments.

## 2. Materials and Experimental Program

### 2.1. Experimental Program

This study was divided into two parts between two labs. The first part of the study investigated the macro-level durability properties, mainly the resistance of concretes to accelerated freeze–thaw of up to 1000 cycles, and the dynamic modulus of elasticity, as per ASTM C666 and ASTM C215, respectively. The second part of this study evaluated the air void parameters by using X-ray CT scan and linear-traverse method, and point count method described in ASTM C457 and in EN 480-11.

### 2.2. Materials and Mixtures

**Figure 1:**
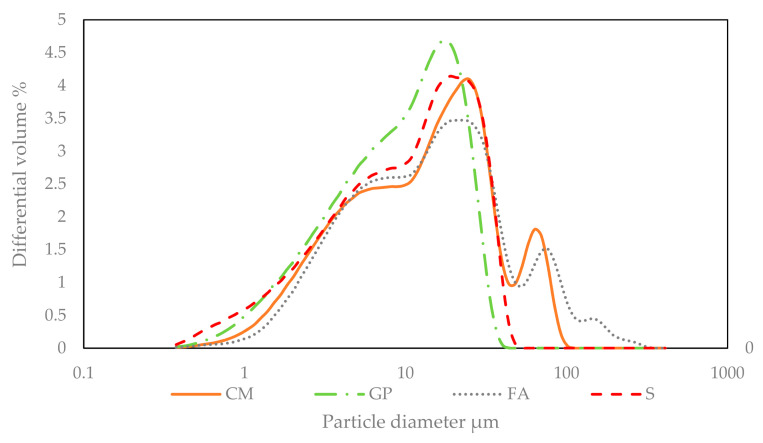
Particle size distribution.

This study evaluated six mixtures for FT resistance and air void system properties: CM (100% cement type I/II), G-20, G-30, G-40 (20%, 30% and 40% cement replacement with glass pozzolan (GP), respectively), FA-30 (30% cement replacement with class F fly ash) and S-40 (40% cement replacement with ground granulated blast-furnace slag). For all mixtures, the cement replacement was per weight. The particle size distribution of cementitious raw materials is shown in Figure 1, as differential volume percent. The median particle size for CM, GP, FA and S, was, respectively, 14, 10, 15 and 11 μm. The chemical composition of the above materials was determined with X-ray fluorescence (XRF), and the loss on ignition was obtained with thermogravimetric analysis (TGA), with values shown in Table 1 and physical properties given in Table 2. The aggregates conformed to specifications by ASTM C33, ASTM C128 and ASTM C127 [28,29,30]. The coarse aggregate was Nova Scotia crushed granite with a maximum nominal size of 19 mm, specific gravity of 2.69 and absorption of 0.7. The fine aggregate was Roanoke sand with specific gravity of 2.63 and absorption of 0.4. The chemical admixtures used were polycarboxylate based for air entraining and water reducing surfactants (complied with Type A and Type F ASTM C494) [31]. All mixture designs had 0.4 water to cementitious content ratio (*w/c*) and followed the NYC-DDC specifications [32] for sidewalk applications, with a focus on durability, with the following standards: Cement content ≤237 kg/m^3^, with no limit on additional SCM content; ratio of fine aggregate to total aggregate content <35%; air content 6 ± 1%; slump 100 ± 25.4 mm. The preparation of the six mixtures followed ASTM C192 [33] specifications and are shown in Table 3. To achieve the performance of durable concrete, the total cementitious content (cement + SCM) was defined at 341 kg/m^3^, and therefore the requirement of maximum cement content of 237 kg/m^3^ was not satisfied for the control mixture with 100% cement (CM) and also for the 20% GP mix (G-20). The air contents and slumps were within the specified limits, by adjusting, respectively, the air-entraining and water-reducing admixtures. The temperature of the concrete was within the expected normal range 22–26 °C. All dry components were mixed for a few minutes until the mixture looked homogenous, and then the water with chemical admixtures was added to the mixer. The specimens were casted into steel molds in two layers to produce homogeneous mixing. At the end specimens were externally vibrated for 5–10 s. Concrete specimens were covered with plastic sheet and placed into a curing room to harden. After 24 h specimens were demolded and returned into the curing chamber with relative humidity of 95–100% and room temperature of 23 °C until the day of testing.

### 2.3. Characterization Methods

The experiments described in this study were carried out to investigate the effects of GP in concrete on porosity and freeze–thaw resistance. All six concrete mixtures were tested for fresh and hardened properties following specific ASTM standards as described in Table 4, where the dimensions of the corresponding cylinder and prism specimens are also noted.

#### 2.3.1. Air Content of fresh Concrete

The field application of this study was to produce durable and sustainable concrete for side-walk construction in New York City (USA), requiring air entrained of 6 ± 1% to enhance cold weather performance, as prescribed by the NYC Department of Design and Construction (DDC). Fresh concrete was tested for air content by the air pressure method as per ASTM C231 [34], and the values are given in Table 3.

#### 2.3.2. Dynamic Modulus of Elasticity (*E*_d_)

The concretes were cured for up to 14 days, removed from the curing room and immediately checked for initial weights. The beam specimens for all six mixtures (three per mixture), were tested for initial dynamic modulus of elasticity *E*_d_*,* obtained by forced resonance method of longitudinal frequency mode as per ASTM C215 [35]. After every 36 freezing and thawing cycles the concrete beams were taken out of the environmental chamber and checked for mass loss and dynamic modulus. The *E*_d_ was calculated as:

(1) $$E_{d}=D\times M\times {\left(n^{\prime }\right)}^{2}$$

where *D* = 4 × (l/b × t) m^−1^ for prisms, *M* is mass of a specimen in kg, *n’* is fundamental frequency in Hz.

#### 2.3.3. Freeze–Thaw Cycles

**Figure 2:**
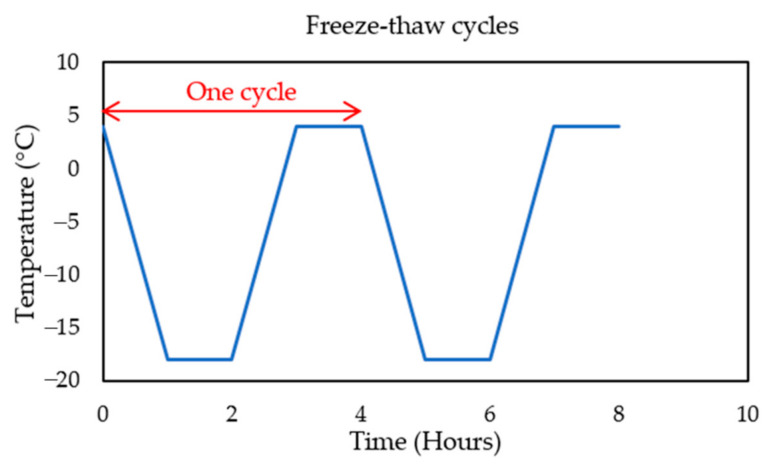
One freeze–thaw cycle.

The testing method followed the standard procedure A from ASTM C666 [36]. After the initial weight check and dynamic modulus of elasticity, the samples were positioned into the trays surrounded by water of up to 3 mm and then placed in the environmental chamber for test-periods of 36 cycles (1000 cycles in total). One cycle lasted 4 h, where the relative humidity was maintained at 50% and the temperature varied between +4 and −18 °C. As it is shown in Figure 2, the temperature decreased linearly from +4 to −18 °C within the first hour. At the second hour the temperature remained constant at −18 °C. In the third hour, the temperature rise from −18 to +4 °C, and at the fourth hour the temperature remained at +4 °C. The level of damage was quantified by the relative dynamic modulus of elasticity and by a durability factor. The beams were visually observed for any scaling effects and macro-cracks.

**Figure 3:**
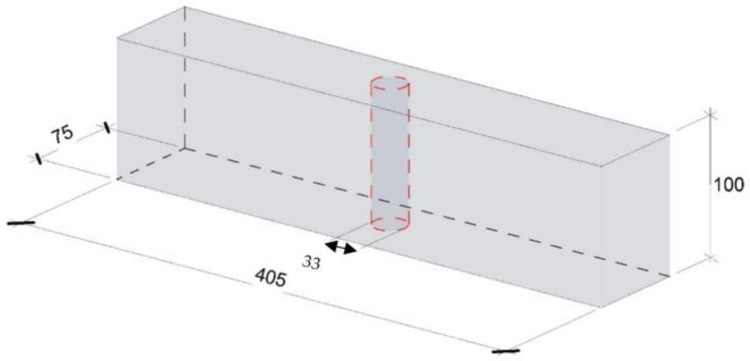
Freeze–thaw beams used for cylinder extraction for X-ray CT scan (mm).

The investigation of micro-cracks induced by FT was performed with X-ray Micro-CT-Scanner (Phoenix Nanotom, Boston, MA, USA), with digital GE DXR detector. One set of samples was used for purposes of evaluating micro-cracks from freezing and thawing. Cores were drilled from the middle of the beams that were exposed to 1000 freeze–thaw cycles. The two cores obtained from each beam were 33 mm in diameter and 50 mm in height, as shown in the Figure 3. The X-ray tube was operated at 130 kV voltage and current of 190 µA. There were 2115 images acquired and the spatial resolution under these conditions was 17.5 µm.

### 2.4. Multi-Scale Characterization of Pore Structure and Micro-Cracks

#### 2.4.1. X-ray Micro-Computerized Tomography (Micro-CT) for Air Void System Analysis

The set of samples for X ray CT scanner and air void analysis were cores drilled from the beams that did not undergo the freeze–thaw exposure; however, they were from the same batch as the freeze–thaw samples, as shown in Figure 3. For each concrete mix, the air void of one core of 20 mm in diameter and 23 mm length was analyzed through a Micro-CT-Scanner (Phoenix Nanotom, Boston, MA, USA), with digital GE DXR detector, there were 1441 projections (2284 × 2304 pixels). In order to minimize the noise, each projection was the resulting average of four radiographs with an exposure of 500 ms. After calibration of the acquired projections with dark and bright field images, the 3D reconstruction was carried out with the software Phoenix datos|x 2.0. During the reconstruction, ring, spot and beam hardening artifacts were corrected. The voxel resolution was 10 µm, and the voltage and current were 140 kV and 170 µA, respectively.

The number of reconstructed images per sample was 2300. In order to eliminate the noise and irregularities of boundary surfaces, images were cropped after original reconstruction. The image analysis was done with open source ImageJ [37] and there were two different approaches applied for calculating the air void content for sake of comparison of results. The first approach was to select 2D images equally spaced through the height of a whole 3D stack, in order to avoid the repetition of the air voids in the vertical direction. Quantification of air void parameters (air content, spacing factor, specific surface) was obtained by using linear-traverse method as per ASTM C457 and EN 480-11 [11,38]. It should be mentioned that both standards require specimens with greater dimensions than the ones used in this study, where the specimens were downsized in order to conveniently use them for both approaches.

**Figure 4:**
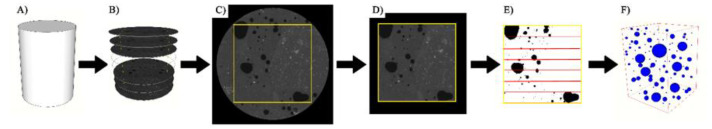
Approach 1: Downsized linear traversed method (2D). (**A**) Extracted core from larger beam, (**B**) Selected images 1 mm apart throughout an entire core specimen, (**C**,**D**) Inscribed square with maximum dimensions within the circle, (**E**) Binary image with applied threshold and traverse lines, and (**F**) square prism as final result.

The calculation of the above mentioned parameters is based on the length of the traverse line which depends on the size of aggregate used in a concrete mixture. The 2D images from the X-ray CT scanner are shown in Figure 4 with grey value (GV) from 0 to 256 (8 bit image). Based on the assumption that all air voids (gel pore, capillary pore, entrained and entrapped air) are not greater than 1 mm, images were taken at 1 mm distance apart along the entire image stack, and were cropped into a square section, hence the linear-traverse method could be applied. The images were converted into binary images and threshold for air voids were defined. The grid of horizontal lines equally distanced from one another was applied on all extracted images (six lines, about 2 mm apart from each other resulting in average traverse line length of 1315 mm). Before calculating all the air void parameters, the following assumptions were made and the rules were applied to all images: (1) Air voids could be categorized as gel pores, capillary pores, entrained air and entrapped air; (2) if the traverse line was intersecting the same void more than once, the closest approximation of single length was made; (3) if the air void was too large and consequently intersected by more than 1 traverse line, only one longest length was considered; and (4) none of the air voids is greater than 1 mm in the Z direction (vertically), if otherwise, in rare cases, when it happened that the entrapped air void was greater than that, the length of the traverse line was accounted for only once, as shown in Figure 4.

**Figure 5:**
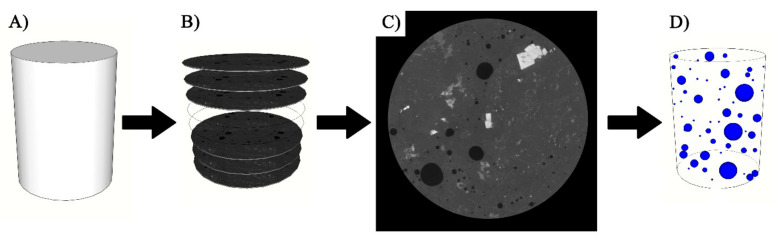
Approach 2: Threshold method of entire stack (3D). (**A**) Extracted core from larger beam, (**B**) Entire image stack, (**C**) A sample of reconstructed image, and (**D**) cylinder prism as final result.

Spacing factor is defined as a relative distance between the voids, which water would have to travel before entering the adjacent void and thereby reducing the pressure. A smaller spacing factor is desirable for FT resistance. The specific surface of air void system is another important parameter and it represents the relative number and size of air voids for a given volume of air (units are mm^−1^). The second approach was by using the entire slice (stack) of 1100 images, converting them into binary images and applying a threshold, black value for voids (0 GV) and white for everything else (256 GV). The area of voids was calculated as per each 2D image, and they were generated throughout the entire height of the image stack (3D). This approach is shown in Figure 5. The disadvantage of this method is that it provides only the air void content, but not the spacing factor nor the specific surface. The results are discussed and compared in a later section.

#### 2.4.2. Mercury Intrusion Porosimetry (MIP)

The pore structure properties were obtained with Micrometrics PoreSizer 9500 mercury intrusion porosimetry (MIP). For the MIP process, a sample is introduced into a chamber, the chamber is evacuated and then the sample is surrounded by mercury. The pressure on the mercury is gradually increased and the maximum pressure of this device is 210 MPa, which corresponds to a minimum pore diameter of about 7 nm and based on a cylindrical pore model. The measurements were carried out in two stages. The first stage was at low-pressure phase from 0 to 0.14 MPa and the second stage was at high pressure running from 0.14 to 210 MPa, as a limit of intrusion. With the increase of pressure, mercury is intruded into the pores that are connected to the surface of the sample. If the pore system is continuous, a pressure may be achieved at which mercury can enter the smallest pore necks of the pore system and penetrate the bulk sample volume. If the pore system is not continuous, mercury may penetrate the pore in the sample by breaking through pore walls. Then extrusion follows the intrusion and it starts by decreasing the pressure to 0.14 MPa. Based on the assumption of cylindrical pore shape model, the relationship between applied pressure *P* (MPa) of the mercury and the diameter of pores intruded by mercury can be calculated by using Washburn’s equation [39].

(2) $$D=\frac{-4\gamma \cos\theta }{P}$$

where γ is surface tension of mercury (0.485 N/m at 25 °C) and θ is the contact angle between the mercury and was taken as 139°.

The samples were cast as small cylinders and they were cured for 100 days, and then sawn into small squares of 10 mm × 10 mm and 3 mm thick. In order to stop hydration, first, samples were immersed in isopropanol five times for 1 min, and in between they were exposed to air for drying. Second, they were immersed in isopropanol for 24 h, and afterwards were placed in a fresh isopropanol and kept in it for seven days. In order to ensure a complete hydration stoppage and to remove isopropanol, the samples were immersed in volatile diethyl ether for 24 h. The samples were dried for 5 min in the oven at 40 °C, and after that they were vacuum dried for 1 h and placed into a desiccator until the day of testing [40,41]. The amount of sample used for each test was between 1.5 and 3 g, because of the different porosity and density of cement pastes for the different SCMs used in the study.

**Figure 6:**
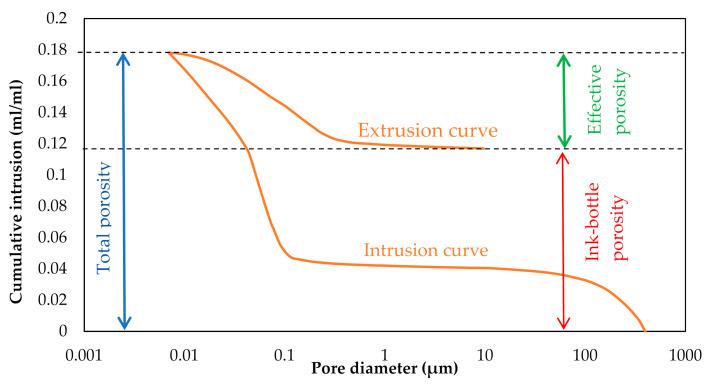
Example of intrusion and extrusion mercury intrusion porosimetry (MIP) curves.

It should be mentioned that MIP does not have the capability of measuring the total pore volume of the paste, which consists of continuous pores, continuous pores with ink bottle neck (mercury cannot get extruded from them), isolated pores and small pores with the diameter smaller than 2 nm (mercury cannot intrude inside). MIP determines the total porosity and effective porosity. Total porosity is calculated as total accessible pore volume (interconnected pores) divided by the bulk volume of the sample, and effective porosity is defined as a volume of mercury intruded under the maximum applied pressure during the second intrusion and divided by the bulk volume of the sample. Consequently, ink-bottle porosity can be calculated by subtracting the effective porosity from total porosity, and it represents the volume of mercury that remains in the pores after extrusion, Figure 6 [42]. Another important parameter that could be measured through MIP is the critical pore size entry diameter determined mathematically from the steepest slope of the cumulative intrusion curve. It should be noted that critical diameter represents only the size of the entry of the pore, not the real diameter of the pore [43].

#### 2.4.3. Nitrogen Adsorption

Nitrogen (N_2_) adsorption has a capability of detecting small pores in materials which is not possible to detect with MIP (from 0.3 to 300 nm) [44]. By combining N_2_ adsorption with MIP it covers the entire pore size range. In this study the nitrogen adsorption tests were performed by using Gemini VII 2390, with a relative pressure (*P/P*_0_) range from 0.05 to 0.999. The relative pressure is defined as equilibrium vapor pressure divided by the saturation vapor pressure. Adsorption and desorption isotherms can be used to study the gel porosity. Condensation of the adsorbate in pores occurs as a function of the pore size. Isotherms are determined experimentally and are usually depicted as the amount adsorbed gas (*Q*_i_) versus the relative pressure (*P/P*_0_) of the gas. In this study, tests were conducted at the age of 100 days as for MIP. N_2_ adsorption was used to determine the size distribution of pores in the range of few nm and to compare the findings with MIP results. Approximately 1 g of a powder sample was used for the analysis. The interpretation of the N_2_ isotherms is based on the mathematical models including the equations of Brunauer-Emmet-Teller (BET) or Barrett–Joyner–Halenda (BJH). The BET theory is based on the multilayer adsorption of gas molecules onto the adsorbent. The pore size distributions can be determined from the adsorption or desorption curves. It is assumed that all pores are filled with N_2_ [45].

### 2.5. Thermogravimetric Analysis (TGA)

The test was performed by increasing the temperature from 40 to 1000 °C at 10 K/minute. In order to quantify the hydration products (more specifically Portlandite or CH) the mass loss was measured according to the following thresholds:

- 40 to 430 °C: Water loss from AFm, AFt (aluminate ferrite mono sulfate, and tri-sulfate, respectively) and C-S-H phases;
- 430 to 550 °C: Water loss from portlandite;
- 550 to 800 °C: Carbon dioxide loss from calcium carbonate minerals.

Portlandite contents were obtained by using the stoichiometry balance of each reaction into account, where WL_Ca(OH)_2__ is weight loss due to water evaporation that can be used to calculate the amount of CH present and molecular masses of CH and water are presented in the ratio (m_Ca(OH)_2__/m_H_2_O_ = 74/18).

Ca(OH)_2, measured_ = WL_Ca(OH)_2__ × m_Ca(OH)_2__/m _H_2_O_ = WL_Ca(OH)_2__ × 74/18 (3)

**Figure 7:**
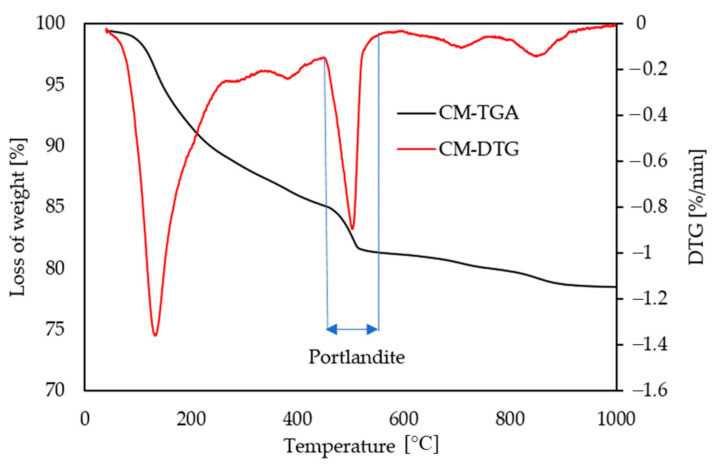
Thermogravimetric analysis (TGA) and DTG versus temperature.

The TG-DTG technique was used to perform tests at 100 days. Netzsch STA 449 F3 Jupiter was used to identify the mass loss for each temperature range. Samples of approximately 55 mg were placed in an aluminum crucible (about 150 µL volume) and exposed under an inert atmosphere of argon. The temperatures ranged from ~40 to 1000 °C at a heating rate of 10 °C/min (see Figure 7). A blank curve, obtained under the same conditions with the same empty aluminum crucible, was systematically subtracted.

## 3. Results and Discussion

### 3.1. Results

#### 3.1.1. Dynamic Modulus of Elasticity (*E*_d_)

**Figure 8:**
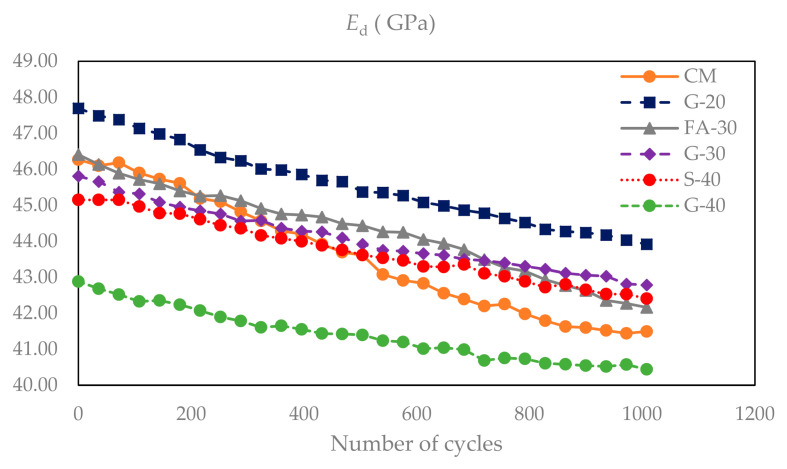
Dynamic modulus of elasticity as a function of number of freeze–thaw cycles.

**Figure 9:**
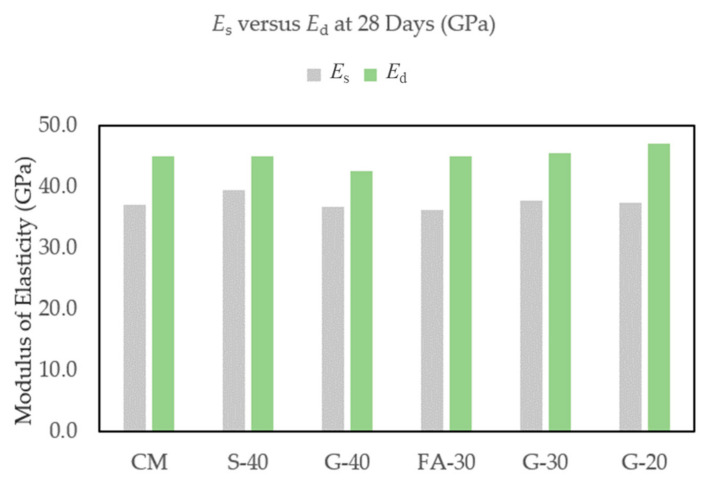
Comparison of *E*_s_ and *E*_d_ at 28 days.

After every 36 FT cycles (six cycles a day), beams were tested for dynamic modulus of elasticity and the results are presented in Figure 8. Initial *E*_d_ for concrete CM was 46.3 GPa. The lowest value was measured for G-40 (42.9 GPa) and the highest value was for G-20 (47.7 GPa). The results for the remaining S-40, G-30 and FA-30 concretes were in between this range (45.1, 45.8, 46.4 GPa, respectively). All concretes with GP and with S showed slight linear decreases in dynamic modulus, while CM and FA-30 concretes had rather stipper decreases in *E*_d_ values. G-40, S-40 and G-30 had, respectively, 5.7%, 6.1% and 6.6% decreases after 1000 cycles, while G-20, FA-30 and CM had slightly higher decreases, but still significant (7.9%, 9.2% and 10.3%, respectively). In Figure 9, values of *E*_d_ at 28 days are compared with corresponding values of static modulus of elasticity (*E*_s_) under compression of cylinder samples and their ratios are calculated showing higher values for *E*_d_ as expected (by ~20% in this study).
$E_{s}=0.83\times E_{d}$for Cement 100%$E_{s}=0.88\times E_{d}$for Slag 40%$E_{s}=0.86\times E_{d}$for Glass 40%$E_{s}=0.80\times E_{d}$for Fly Ash 30%$E_{s}=0.83\times E_{d}$for Glass 30%$E_{s}=0.80\times E_{d}$for Glass 20%

#### 3.1.2. Freeze–Thaw Cycling

**Figure 10:**
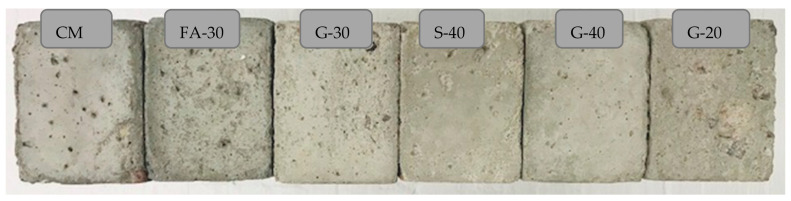
Visual observation of scaling and deterioration.

**Figure 11:**
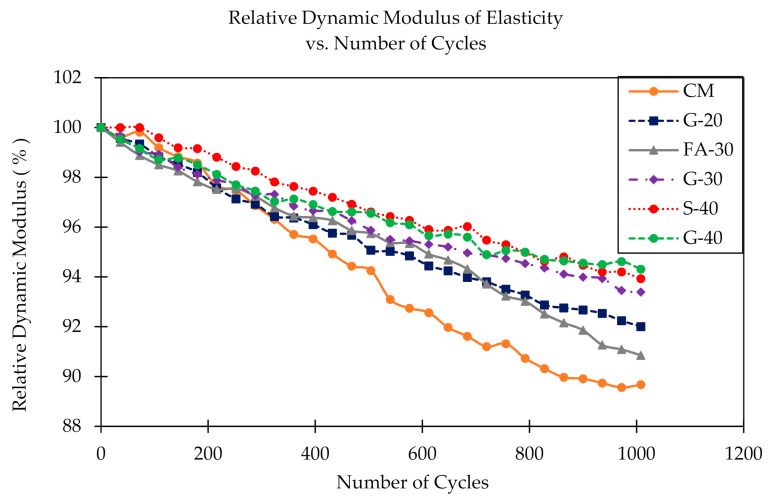
Relative dynamic modulus after freeze–thaw cycling.

The visual images of the cross sections of the FT samples are shown in Figure 10. In Figure 11 the results of relative dynamic modulus of concretes CM, G-20, FA-30, G-30, S-40 and G-40 are presented. From the graph, it can be seen that all concretes showed high resistance to freezing and thawing at 1000 cycles, since their relative dynamic modulus is in the range of 90% to 94%, which is significantly higher than a suggested minimum value of 60% for concretes to be FT resistant [36]. The highest FT resistance was recorded for S-40 and G-40, which showed nearly equal values (93.9% and 94.3%, respectively). In Table 5, the durability factor and mass loss are presented. Except for CM, (0.897), the durability factor was above 0.9 for the rest of the mixtures. Mass loss was less than 1% for all the mixtures with GP and S, while the mass loss for FA and CM exceeded slightly 1% which is still insignificant. The specimens were visually observed for surface damage, cracks, aggregate pop outs, flaking and corner chipping [46], and there were no visible cracks, but some degree of scaling and corner chipping of CM, FA-30 and G-20, as can be observed in the Figure 10.

**Figure 12:**
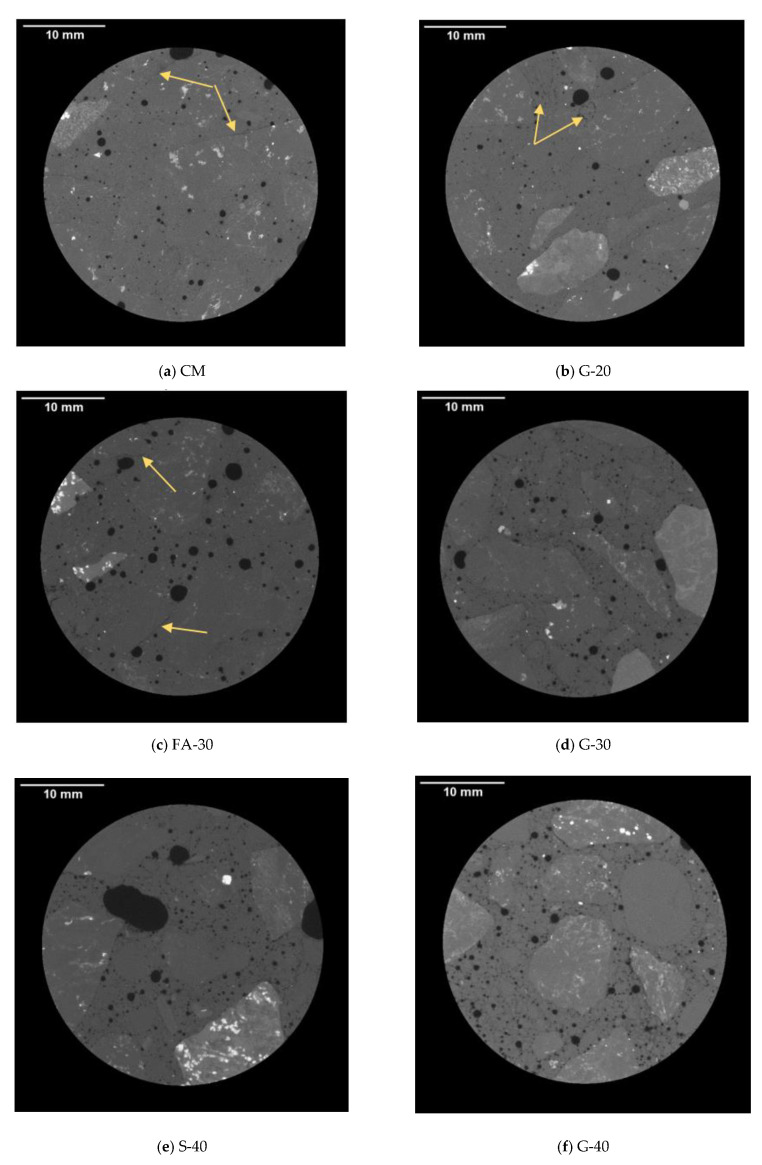
Micrographs for micro-cracking analysis at 1000 freeze–thaw (FT) cycles.

In Figure 12, micrographs are shown for micro-cracking analysis at 1000 FT cycles. There were no clearly visible cracks. For all concretes, it seems that there was an increased micro-porosity within the interfacial transitional zone (ITZ) of paste and aggregate, and at the spatial resolution of 17.5 µm it cannot be clearly seen whether there was a micro-crack, which perhaps closed due to the self-healing of concretes. In Figure 12b for G-20 there were some micro-cracks; however, they could be a result of applied pressure during drilling the core sample from the beam. It was noted from the grey value (GV) images, as shown in the Figure 12c, that FA-30 seems to be the darkest. It is believed to be related to the density of concrete, which in the case of FA was the lowest value among the six concrete mixtures. According to the Beer–Lambert law, it is known that the attenuation of the X-ray depends on the density of a material (higher density of a material may result in lighter GV images), beam intensity and the thickness of the penetrated material [47,48].

#### 3.1.3. Air Void Analysis of Hardened Concrete Using X-ray Computed Tomography

**Figure 13:**
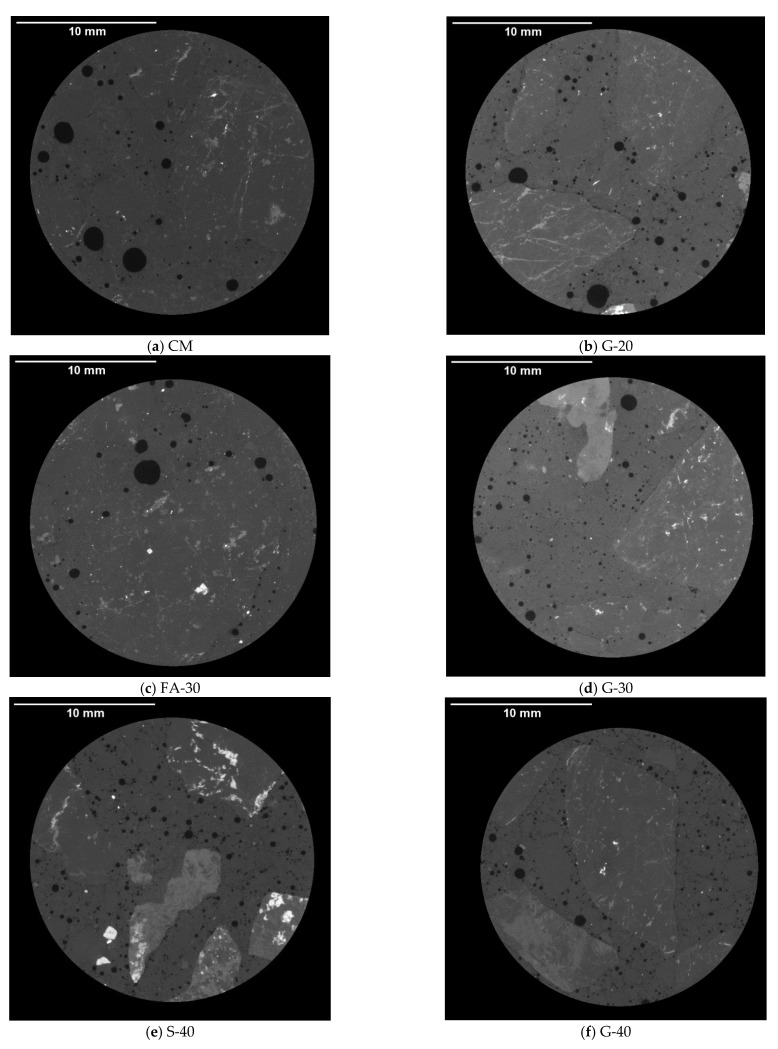
Micrographs for air void analysis.

**Figure 14:**
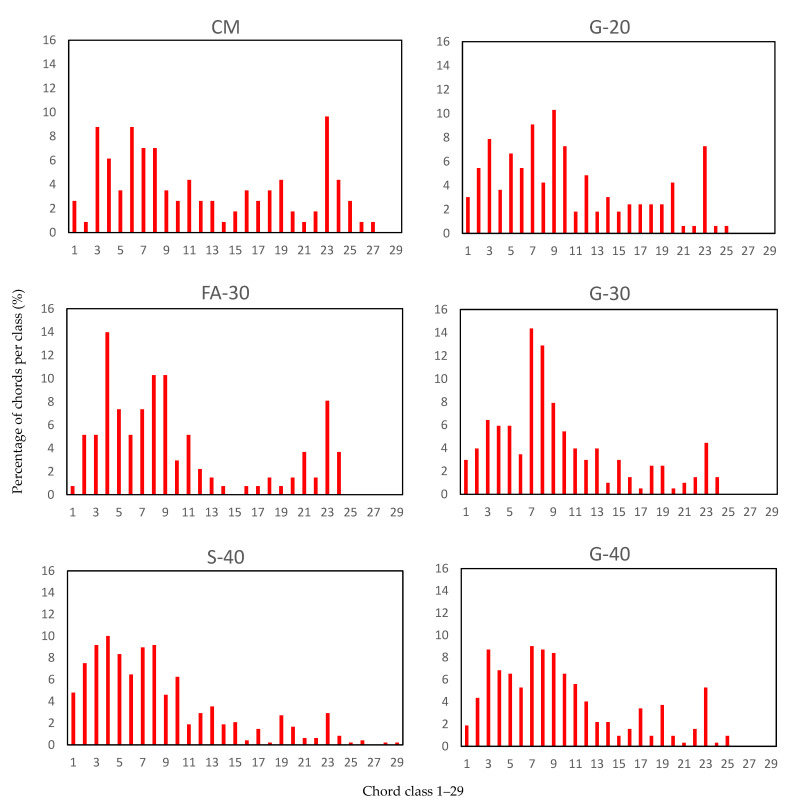
Classification of the air voids in 29 classes from 0 to 4000 μm (see Appendix A).

The starting point for air void analysis of hardened concrete was the question after the hydration process is completed, whether concrete still contains the same amount of entrained air determined from the fresh concrete mixtures by air-pressure method. Air-content obtained from fresh concrete was in the range of 5.2% to 6.2% and it is presented in the Table 6. From Figure 13, the micrographs obtained by Micro-CT-scanner, show that concretes with GP and S had smaller air voids and more uniformly dispersed than concretes with FA and CM. In Figure 14, classification of the air voids is summarized in 29 classes (see Appendix A). It was noted that air voids were scattered within all the classes for CM, while for other concretes more than 70% of air voids were up to 200 µm (classes 1 through 13). Except for CM and G-20, the rest of concretes showed more than 50% of air voids up to 100 µm (classes 1 through 8). Air content was highest for S-40 and G-40 (5.4% and 4.3%, respectively). The rest of the concretes showed air void contents between 2% and 3%. The spacing factor is reported in Table 7, denoted as L (mm), and shows that all concretes had smaller than 0.3 mm except for CM (0.452 mm). Both S-40 and G-40 had the smallest spacing factor of less than 0.2 mm (0.15 and 0.19 mm, respectively). In regards to specific surface, α, CM had only 11.9 mm^2^/mm^3^, while S-40 had 26.2 mm^2^/mm^3^. Both G-20 and FA-30 had ~21 mm^2^/mm^3^, while G-30 and G-40 measured ~24 mm^2^/mm^3^. The amount of paste, P, was ~21% for all concretes. The linear traverse method, A, showed slightly higher percent air void content than the approach completely based on Threshold of an entire stack (see Table 6). However, the results from these two methods are very similar except for G-20. In comparison to air content of fresh concretes, air void content of hardened concrete was significantly lower for all concretes, except for S-40 and G-40 (12.9% and 25.8% lower, respectively).

In Table 7, α is specific surface of the air voids, *P* is paste content by volume calculated from the mix design proportions, *A* is air void content (%) calculated by the linear traverse method, *R* represents the ratio of paste and air void content (*P/A*), L is spacing factor, and formulas are given in Appendix A.

#### 3.1.4. Mercury Intrusion Porosimetry (MIP)

**Figure 15:**
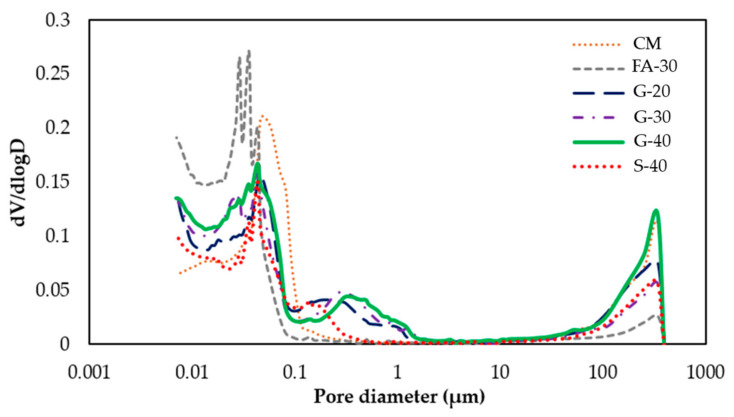
Critical pore entry diameter.

The total porosity of connected pores including micro, meso and macro pores was determined together with effective porosity, ink bottle porosity and pore connectivity (see Table 8). The volume of total porosity was found to be the lowest for S-40 (~14%), while CM and FA-30 had porosities of 17.8% and 17.6%, respectively. Cement pastes with GP reported higher total porosities than the other cement pastes, with values for G-20, G-30 and G-40 as 19.1%, 18.5% and 21.3%, respectively, showing that the addition of GP resulted in increase in porosity. For G-40 the increased in porosity is ~20% higher compared to CM and ~53% higher in relation to S-40. The ink-bottle porosity for FA-30 (10.3%) and S-40 (9.8%) showed to be lower than for the reference paste CM (11.7%); however, pastes G-20, G-30 and G-40 showed increases of 12.8%, 11.9% and 13.9%, respectively. Effective porosity for the reference paste CM was 6.1% and higher for FA-30 (7.4%), and G-20, G-30 and G-40 (6.3%, 6.6% and 7.4%, respectively). Figure 15 shows the differential intruded volume (critical pore entry diameter) for the six pastes.

From the MIP differential curve and pore size distribution, for Portland cement pastes there are two different corresponding pore systems [49,50]. As it is shown in Figure 15 the first peak of cement pastes has a pore diameter between 0.01 and 0.1 µm that corresponds to a threshold pore diameter of a gel pore system, while the second diameter corresponds to a threshold diameter of a capillary pore system, and it is between 0.1 and 1 µm [51]. It can be noticed that CM and G-20 have the same critical pore diameter of 0.0465 µm, while by replacing Portland cement with a percentage of GP above 20%, the critical pore size diameter slightly decreased to 0.043 µm for G-30 and 0.0435 µm for G-40. The addition of FA and S also resulted in a decrease of critical pore diameter (0.035 and 0.0433 µm).

It was also observed that the pore volume between 10 nm and 100 nm increased by adding GP and FA, which may indicate an increased gel porosity [49]. These results show an indication of a refined pore structure and reduced percolation throughout the pore system when more percentage of cement is replaced with GP. Consistent with these results, in [52,53] the authors also reported reduced porosity by adding more GP, while they also noticed the decrease in critical pore diameter and refined pore structure with higher percentage of cement replacement with GP. In Table 8, the test results show that for all SCMs the bulk density decreased in comparison to the reference CM paste. In Table 8 the pore structure properties are given. The pore connectivity for CM was 34.4%, with about the same range for pastes with GP; the lowest was for S-40 (29.6%) and the highest for FA-30 (41.7%).

#### 3.1.5. Nitrogen Adsorption

**Figure 16:**
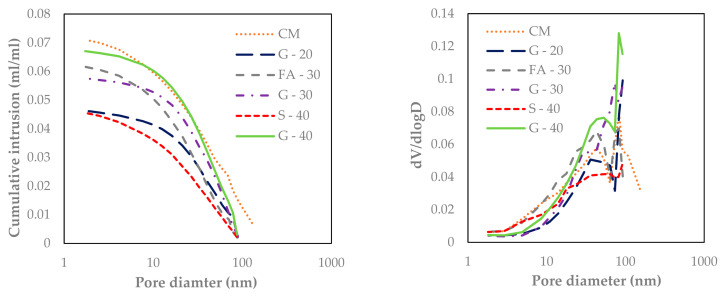
Pore size distribution (**left**) and differential curves (**right**) of cement pastes at 100 days.

By coupling MIP and N_2_ adsorption, the entire pore size range is covered. The results are reported in Figure 16. The pores detected by N_2_ adsorption were in the range from 2 to 152 nm for the reference paste CM. From the differential curves (right, Figure 16) it can be noticed that there are two peaks for all pastes: S-40 showed the smallest, barely noticeable peaks, whereas G-40 showed the highest peaks, the first at 53 nm and the second at 82 nm diameter, and this corresponds with aforementioned gel pores within the range of 10–100 nm discussed in Section 3.1.4. It is observed that G-40 shows the highest volume in gel porosity, then followed by the decreasing volumes for G-30 and G-20, respectively. These findings showed an overlap of the peaks for the critical pore diameters between 10 and 100 nm which are in agreement with the results from MIP.

#### 3.1.6. Thermogravimetric Analysis (TGA)

**Figure 17:**
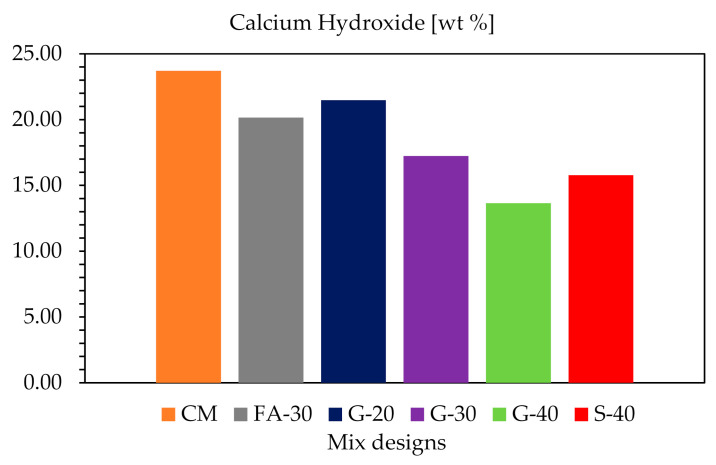
Calcium hydroxide for all pastes at 100 days.

The temperature range for calculating CH content is considered to be from 430 to 550 °C, as used in this study. The CH contents obtained for all six mixes are shown in Figure 17. As expected CM has the highest amount of CH (~24%) because it has the highest amount of cement. Pastes with GP (G-20, G-30 and G-40) show a decreasing trend in CH with increase of cement replacement by GP (about 21%, 17% and 14%, respectively). When G-30 is compared with FA-30 (~20%) and G-40 with S-40 (~16%), pastes with GP showed less portlandite than their comparable references which is a clear indication of enhanced pozzolanic reactivity.

### 3.2. Discussion

Replacing cement with up to 40% by weight of GP had a clear impact on the dynamic modulus of elasticity (*E*_d_) and freeze thaw resistance of concrete, as presented in the previous Section 3.1.1 and Section 3.1.2. Initially G-20 showed the highest values for *E*_d_, but the lowest decrease after 1000 FT cycles. In contrast, G-40 showed initially lower *E*_d_ but smaller reduction at the final stage. This is in an agreement with compressive strength and static modulus of elasticity results from previous research work by Krstic and Davalos [54,55,56], where up to 28 days, concretes with higher cement content showed better performance due to primary hydraulic reactions. Among the three mixes with GP, the mix with lesser glass content, G-20, reached higher early strength up to 28 days, but due to higher pozzolanic reactivity both G-30 and G-40 showed higher strengths at 56 days, and all three reached comparable values at 90 days. These trends correspond with the present results for relative dynamic modulus and durability factor. As it is shown in Figure 9, the *E*_d_ is greater than *E*_s_ for about 20%, which is partly due to the differences in specimen geometry and the nature of static and dynamic loads. The durability factor was above 90% for all six concretes, and indicates that there was insignificant deterioration of specimens, due in part to entrained air of ~6%. Judging from the visual inspection of the surfaces and from X-ray CT scans, even if some small micro-cracks occurred, which is likely, they probably self-healed after some time.

It is worth mentioning that the difference in air content of fresh concrete and air void content of hardened concrete was more evident for CM, FA-30, G-20 and G-30. As shown, G-40 and S-40 had the smallest decrease in air void content. There could be many factors influencing this reduction, such as type of SCM used, conditions during the placing of concrete (temperature, compaction, vibration) and environmental exposure. Since the hardened concrete that was tested for air void content was about four years old, perhaps over time hydration products filled up the voids. It was observed that GP and S had similar mean particle size *d*_50_ of 10 and 11 µm and they are both angular in shape. Probably because of their larger specific surface than CM and FA, as 40% cement replacements both GP and S serve as nucleation for air bubbles.

The linear traverse method showed slightly higher air void content than the approach solely based on threshold of an entire stack. The difference in results could be due to various reasons, a slightly different geometry of a sample considered for calculating air content, or in the case of a linear traverse method it is possible that human error can likely occur. The Threshold method of an entire stack did not account for any voids smaller than 50 µm, while the linear traverse method did, as per Figure 14. The linear traverse method applied through X-ray CT scan is a tedious procedure and it is difficult to make a judgement as to whether the air voids are smaller than 50 µm, so it is easier to account for all of them. Therefore, it is reasonable that the linear traverse method gives a slight overestimation of the air void content. In terms of the threshold method of the entire stack, G-20 showed the biggest difference with respect to the linear traverse method. This is most likely due to applying the same threshold on all samples; however, it was noted from the grey value (GV) images (Figure 13) that there was a slight difference among the mixes. According to the Beer–Lambert law, it is known that the attenuation of the X-ray depends on the density of a material (higher density of a material may result in lighter GV images), beam intensity and the thickness of the penetration [47,48]. From the air void analysis it was also shown that G-40 and S-40 reported the most desirable parameters prescribed for freeze–thaw resistant concrete as measured by spacing factor smaller than 0.2 mm and specific surface greater than 24 mm^2^/mm^3^ [8]. The distribution of pores for these two mixes had the majority of pores being less than 200 µm, which led to high specific surfaces.

While MIP showed slightly increased total porosity for pastes with higher cement replacement with GP, the differential curves indicated an increase of gel pore volume. The N_2_ adsorption method had also confirmed the increase of volume of gel porosity with adding more GP. The TGA results substantiated as well that there is more gel formation (C-S-H) by increasing the cement replacement with GP, in comparison to their references. The findings presented in this study indicate that improved freeze–thaw resistance and refined pore structure of concrete are probably due to the filling effect and pozzolanic effect of GP due to CH consumption for creating more C-S-H.

While it is widely recognized that air-entrainment improves the freeze–thaw resistance of concrete in general, it was noted that the mixes with the highest cement replacement of 40% by S and GP showed the highest durability factor and least mass loss of concrete. These concretes also showed the most desirable air void parameters of hardened concrete (air void content, spacing factor and specific surface). According to this study 40% GP replacement showed that it is as successful as 40% S to improve the FT resistance of concrete, by maintaining a recommended spacing factor and specific surface which are the most important parameters.

## 4. Conclusions

In this study we investigated the freeze–thaw (FT) resistance of concretes containing cement replacement by GP of up to 40% by weight, and comparing the performance with other commonly used SCMs. Macroscopic properties such as air content of fresh concrete, static and dynamic modulus of elasticity, relative dynamic modulus, mass loss and durability factor were obtained through standard ASTM methods. The internal micro-cracks of concrete subjected to freeze–thaw, and the air void analysis of hardened concrete that was not exposed to environmental effects, were evaluated with X-ray computed tomography, and the data was processed with ImageJ. In order to explain the observed behavior in concretes, the pore structure of the pastes was characterized through MIP and N_2_ adsorption analyses.

Based on the combined macro and micro-evaluations of concretes and cement pastes with GP in this study, the following conclusions can be drawn:

1. The samples were simultaneously tested for freeze–thaw cycles and dynamic modulus, which decreased continuously up to about 10% at 1000 freeze–thaw cycles, indicating deterioration of the samples.
2. A durability factor of above 90%, and mass loss of ~1% for all concretes except for CM (1.6%), indicated improved freeze–thaw resistance with increased of cement replacements by GP, due to its pozzolanic activity, and perhaps consuming more CH for C-S-H formation.
3. Using GP as an alternate SCM, serves as nucleation for air bubbles due to its angularity and finer particle size than CM, and consequently its larger specific surface area. Spacing factor decreases and specific surface increases with higher cement replacement by GP, which are favorable for FT resistance.
4. Based on MIP and N_2_, higher replacement of cement by GP results in slightly higher total porosity, but also more refined pore structure. By increasing the GP content, the pore size gets more refined (gel pores and pore size distribution increase).
5. Calcium hydroxide (CH) was quantified by TGA. Even though binary systems containing SCMs had less cement content for creating CH, when G-30 was compared to FA-30 and G-40 to S-40, results showed that CH was consumed more in the systems when adding higher contents of GP, leading to C-S-H formation. G-40 showed the least portlandite present at all times, which clearly indicates the pozzolanic reactivity of GP.
6. The air void analysis by X-ray micro-tomography coupled with ImageJ can be successfully used to evaluate the microstructure of cementitious materials. It is a nondestructive method, and it can provide 3D information that is especially useful for air void analysis. This method requires minimum sample preparation, unlike the standard method ASTM C457; however, it has a limitation on sample size.
7. Micro-cracks after 1000 FT cycles were almost unnoticeable. This implies a good FT resistance which is in agreement with the overall analyses results, and/or it could mean that there were very small cracks which coalesced probably due to self-healing of concrete over time.

## Figures and Tables

**Table 1:** Chemical compositions of raw materials obtained through XRF.

Chemical Composition	Glass Pozzolan (GP)	Fly Ash Class F (FA)	Slag (S)	Portland Cement (PC)
SiO_2_, %	72.5	47.58	38.00	20.2
Na_2_O, %	13.7	1.5	0.32	0.19
CaO, %	9.7	5.54	39.84	61.9
Al_2_O_3_, %	0.4	26.42	7.52	4.7
MgO, %	3.3	0.9	10.54	2.6
K_2_O, %	0.1	1.9	0.38	0.82
Fe_2_O_3_, %	0.2	12.19	0.31	3.0
SO_3_, %	0.1	1.08	0.16	3.9
Total alkalis Na_2_O + 0.658K_2_O, %	13.77	2.75	0.6	0.73
LOI, %	0.4	2.5	1.2	1.5

**Table 2:** Physical properties of raw materials.

Physical Properties	Portland Cement	Glass Pozzolan	Fly Ash Class F	Slag
% Passing #325 Mesh %	90.00	100	81.50	99
Specific Gravity	3.15	2.46	2.52	3
Median Particle Size (micron)	14	10	15	11
Strength Activity Index, 28d, %	-	102	-	-
Water Requirement, % of Control %	-	97	-	-
Soundness %	-	0.05	-	-
Moisture Content %	-	0.10	-	-
Brightness %	-	80–87	-	-
Specific Area cm^2^/g	-	4840	-	-

**Table 3:** Mix designs of concretes.

Ingredients	CM	G-20	FA-30	G-30	S-40	G-40
Cement type I/II, kg/m^3^	341	273	237	237	205	205
Glass pozzolan, kg/m^3^	-	68	-	104	-	137
Class F fly ash, kg/m^3^	-	-	104	-	-	-
Slag, kg/m^3^	-	-	-	-	137	-
Coarse aggregate kg/m^3^	1196	1193	1171	1187	1196	1187
Fine aggregate, kg/m^3^	640	631	631	631	632	630
Water content, kg/m^3^	138.2	138.2	138.8	139.4	140	140.6
Water-reducing admixture, ml/m^3^	660	695	735	775	815	850
Air-entraining admixture, ml/m^3^	620	660	1150	735	775	810
Water-cement ratio (*w*/*c*)	0.41	0.41	0.41	0.41	0.41	0.41
Slump, in. mm	115	100	100	100	115	100
Air content, %	5.9	5.2	5.6	5.2	6.2	5.8
Temperature, °C	24	24	26	23	22	22

**Table 4:** Testing methods and specimens.

Characterization of Raw Materials	Standard	Specimen	
Particle size distribution	-	powder	
X-ray fluorescence	-	powder	
**Test Methods of Fresh Concrete**			
Air content pressure method	ASTM C 231	-	-
Slump	ASTM C 143	-	-
Temperature of fresh concrete	ASTM C 1064	-	-
**Test Methods of Hardened Concrete**			
Static modulus of elasticity	ASTM C 469	Cylinder 100 mm × 200 mm	
Dynamic modulus of elasticity	ASTM C215	Prism 75 mm × 100 mm × 405 mm	
Freeze–thaw resistance test	ASTM C 666	Prism 75 mm × 100 mm × 405 mm	
Air-void analysis (modified) CT	ASTM C 457	Cylinder 20 mm × 23 mm	
Micro-cracking analysis CT	-	Cylinder 33 mm × 50 mm	
Mercury intrusion porosimetry	-	Prism 10 mm × 10 mm × 3 mm	
Nitrogen adsorption	-	powder	
Thermogravimetric Analysis	-	powder	

**Table 5:** Durability properties.

	CM	G-20	FA-30	G-30	S-40	G-40
Durability factor	0.90	0.920	0.91	0.93	0.94	0.94
Mass Loss %	1.58	0.75	1.01	0.6	0.56	0.52

**Table 6:** Summary of air void content obtained with different methods.

%	CM	G-20	G-30	G-40	S-40	FA-30
Air voids-Traverse method	3.0	2.3	2.5	4.3	5.4	2.4
Air voids-Threshold	2.9	1.1	2.2	3.9	4.5	1.8
Air content-Air-pressure	5.9	5.2	5.2	5.8	6.2	5.6

**Table 7:** Air void parameters.

	CM	G-20	G-30	G-40	S-40	FA-30
α (1/mm)	11.9	20.7	24.2	23.8	26.2	21.3
*P* (%)	20.7	20.8	20.9	21.0	20.9	21.0
*A* (%)	3.0	2.3	2.5	4.3	5.4	2.4
*R*	6.9	8.9	8.4	4.9	3.9	8.8
*L* (mm)	0.45	0.29	0.24	0.19	0.15	0.28

**Table 8:** Pore structure properties.

	CM	G-20	FA-30	G-30	S-40	G-40
Bulk density (%)	1.79	1.66	1.74	1.64	1.73	1.55
Total Porosity (%)	17.8	19.1	17.6	18.5	13.9	21.3
Ink-Bottle Porosity (%)	11.7	12.8	10.3	11.9	9.8	13.9
Effective Porosity (%)	6.1	6.3	7.4	6.6	4.1	7.4
Critical Pore Diameter (µm)	0.0465	0.0465	0.035	0.043	0.0433	0.0435
Pore Connectivity (%)	34.4	33.0	41.7	35.7	29.6	34.6

## Data Availability

The data presented in this study are available on request from the corresponding author.

## Appendix A

Air void analysis

(A1) $$\alpha =\frac{4\times N}{T_{a}}$$

(A2) $$A=\frac{T_{a}\times 100}{T_{t}}$$

(A3) $$4.342L=\frac{3}{\;\alpha \;}\left[1.4{\left(1+\frac{P}{A}\right)}^{\frac{1}{3}}-1\right]\;P/A\;>\;4.342$$

$T_{a}$–traverse length throughout air, *N*–total number of air void intersected, $T_{t}$–total length of traverse line.

**Table A1:** Classification of the pores by size (μm).

Class	From	To
1	0	10
2	15	20
3	25	30
4	35	40
5	45	50
6	55	60
7	65	80
8	85	100
9	105	120
10	125	140
11	145	160
12	165	180
13	185	200
14	205	220
15	225	240
16	245	260
17	265	280
18	285	300
19	305	350
20	355	400
21	405	450
22	455	500
23	505	1000
24	1005	1500
25	1505	2000
26	2005	2500
27	2505	3000
28	3005	4000
29	>	4005

## References

[1] Pawlowicz J. (2019). Evaluation of Air Entraining Behaviour in Concrete Using Computer Aided Methods on Hardened Samples. Master’s Thesis.

[2] Hanjari K.Z., Utgenannt P., Lundgren K. (2011). Experimental study of the material and bond properties of frost-damaged concrete. Cem. Concr. Res..

[3] Neville A.M. (2011). Properties of Concrete.

[4] Shang HS S.Y. (2006). Experimental study of strength and deformation of plain concrete under biaxial compression after freezing and thawing cycles. Cem. Concr. Res..

[5] Keleştemur O., Yildiz S., Gökçer B., Arici E. (2014). Statistical analysis for freeze-thaw resistance of cement mortars containing marble dust and glass fiber. Mater. Des..

[6] Gagne R. (2016). Science and Technology of Concrete Admixtures.

[7] Whiting D., Nagi M. (1998). Control of Air Content in Concrete.

[8] Saucier F., Pigeon M., Cameron M. (1991). Air-void stability-part V: Temperature, general analysis, and performance index. ACI Mater. J..

[9] Peterson K., Sutter L., Radlinski M. (2009). The practical of application a flatbed scanner for air-void characterization of hardened concrete. J. ASTM Int..

[10] Peterson K.W., Anzalone G.C., Nezami S., Oh C.Y.S., Lu H. (2016). Robust Test of the Flatbed Scanner for Air-Void Characterization in Hardened Concrete. J. Test. Eval..

[11] ASTM C 457/C 457M-12 (2013). Standard Test Method for Microscopical Determination of Parameters of the Air-Void System in Hardened Concrete 1.

[12] Kim K.Y., Yun T.S., Choo J., Kang D.H., Shin H.S. (2012). Determination of air-void parameters of hardened cement-based materials using X-ray computed tomography. Constr. Build. Mater..

[13] Lanzón M., Cnudde V., De Kock T., Dewanckele J. (2012). X-ray microtomography (μ-CT) to evaluate microstructure of mortars containing low density additions. Cem. Concr. Compos..

[14] Kosmatka S.H., Wilson M.L. (2014). Design and Control of Concrete Mixtures.

[15] Nedeljković M., Li Z., Ye G. (2018). Setting, strength, and autogenous shrinkage of alkali-activated fly ash and slag pastes: Effect of slag content. Materials (Basel).

[16] Huntzinger D.N., Eatmon T.D. (2009). A life-cycle assessment of Portland cement manufacturing: Comparing the traditional process with alternative technologies. J. Clean. Prod..

[17] Chatham House (2018). Making Concrete Change: Innovation in Low-Carbon Cement and Concrete. https://www.chathamhouse.org/2018/06/making-concrete-change-innovation-low-carbon-cement-and-concrete.

[18] U.S. Geological Survey (2018). Cement Statistics and Information. http://minerals.usgs.gov/minerals/pubs/commodity/cement/.

[19] Kamali M., Ghahremaninezhad A. (2015). Effect of glass powders on the mechanical and durability properties of cementitious materials. Constr. Build. Mater..

[20] Sheikh V. (2018). Limited Availability of Cementitious Materials Could Impact the Value Chain. Appl. Sci. Sustain. Coal Ash.

[21] Roston E., Migliozzi B. (2015). Obama’s EPA Rule Is Redrawing the U.S. Coal Map. Bloomberg New Energy Finance-US Coal Retirements Database. http://www.bloomberg.com/graphics/2015-coal-plants/.

[22] Hodge T. (2015). Power Generation from Coal and Natural Gas Expected to Temporarily Converge This Spring. U.S. Energy Information Administration. http://www.eia.gov/todayinenergy/detail.cfm?id=21232.

[23] Ober J.E., U.S. Geological Survey (2017). Mineral Commodity Summaries. https://www.usgs.gov/centers/nmic/arsenic-statistics-and-information#mcs.

[24] Kamali M., Ghahremaninezhad A. (2016). An investigation into the hydration and microstructure of cement pastes modified with glass powders. Constr. Build. Mater..

[25] Kaminsky A., Krstic M., Rangaraju P., Hamou A.T., Thomas M. (2020). Ground-Glass Pozzolan for Use in Concrete, Members of ASTM Subcommittee C09.24 summarize industry context behind new ASTM standard specification. Concr. Int..

[26] Shayan A. Value-added utilization of waste glass in concrete. Proceedings of the IABSE Symposium.

[27] Omran A., Tagnit-hamou A. (2016). Performance of glass-powder concrete in field applications. Constr. Build. Mater..

[28] ASTM C33 (2015). Standard Specifications for Concrete Aggregates.

[29] ASTM C128 (2015). Standard Test Method for Density, Relative Density (Specific Gravity), and Absorption of Fine Aggregate.

[30] ASTM C127 (2015). Standard Test Method for Density, Relative Density (Specific Gravity), and Absorption of Coarse Aggregate.

[31] ASTM C494 (2015). Standard Specification for Chemical Admixtures for Concrete.

[32] NYC (2015). D.D.C. of Standard Highway Specifications Volume I of II.

[33] ASTM C192 (2015). Standard Practice for Making and Curing Concrete Test Specimens in the Laboratory.

[34] ASTM C231 (2015). Standard Test Method for Air Content of Freshly Mixed Concrete by the PressureMethod.

[35] ASTM C215 (2015). Standard Test Method for Fundamental Transverse, Longitudinal, and Torsional Resonant Frequencies of Concrete Specimens.

[36] ASTM C666 (2015). Standard Test Method for Resistance of Concrete to Rapid Freezing and Thawing.

[37] Rueden C.T., Schindelin J., Hiner M.C., DeZonia B.E., Walter A.E., Arena E.T., Eliceiri K.W. (2017). ImageJ2: ImageJ for the next generation of scientific image data. BMC Bioinform..

[38] BS EN 480-11:2005 (2005). Admixtures for Concrete, Mortar and Grout- Test Methods Part 11: Determination of Air Void Characteristics in Hardened Concrete.

[39] Kaufmann J. (2010). Pore space analysis of cement-based materials by combined Nitrogen sorption-Wood’s metal impregnation and multi-cycle mercury intrusion. Cem. Concr. Compos..

[40] Zhang J., Scherer G.W. (2011). Comparison of methods for arresting hydration of cement. Cem. Concr. Res..

[41] Scrivener K., Snellings R., Lothenbach B. (2016). A Practical Guide to Microstructural Analysis of Cmenetitious Materials.

[42] YE G. (2003). Experimental Study and Numerical Simulation of the Development of the Microstructure and Permeability of Cementitious Materials. Ph.D. Thesis.

[43] Diamond S. (2000). Mercury porosimetry. An inappropriate method for the measurement of pore size distributions in cement-based materials. Cem. Concr. Res..

[44] Gerhardt R. (1988). As Review of Conventional and Non-Conventional Pore Characterization Techniques. MRS Proc..

[45] Barrett E.P., Joyner L.G., Halenda P.P. (1951). The determination of pore volume and area distributions in porous substances. I. Computations from nitrogen isotherms. J. Am. Chem. Soc..

[46] Nassar R., Soroushian P. (2012). Strength and durability of recycled aggregate concrete containing milled glass as partial replacement for cement. Constr. Build. Mater..

[47] Roels S., Vandersteen K., Carmeliet J. (2003). Measuring and simulating moisture uptake in a fracturedporous medium. Adv. Water Resour..

[48] Šavija B., Luković M., Schlangen E. (2017). Influence of cracking on moisture uptake in strain-hardening cementitious composites. J. Nanomech. Micromech..

[49] Cook R.A., Hover K.C. (1999). Mercury porosimetry of hardened cement pastes. Cem. Concr. Res..

[50] Feldman F. (1984). Pore structure damage in blended cements caused by mercury intrusion. J. Am. Ceram. Soc..

[51] Yu Z. (2015). Microstructure Development and Transport Properties of Portland Cement-fly Ash Binary Systems. Ph.D. Thesis.

[52] (2015). Hongjian Du and Kiang Hwee Tan Transport Properties of Concrete with Glass Powder as Supplementary Cementitious Material. ACI Mater. J..

[53] Du H., Tan K.H. (2014). Waste glass powder as cement replacement in concrete. J. Adv. Concr. Technol..

[54] Krstic M., Davalos J.F. (2019). Field application of recycled glass pozzolan for concrete. ACI Mater. J..

[55] Krstic M. (2020). Macro- and Micro-structure Evaluations and Field Applications of Concrete with Recycled Glass. Ph.D. Thesis.

[56] Krstic M., Davalos J.F., Miguel A., Dirk Schlicke F.B., Jędrzejewska A. (2018). Macro-and Micro-structure Evaluations of Recycled Post-consumer Glass Cementitious Material for Concrete. Proceedings of the Interdisciplinary Approaches for Cement-Based Materials and Structural Concrete: Synergizing Expertise and Bridging Scales of Space and Time.

